# Preparation of β-cyclodextrin/polysaccharide foams using saponin

**DOI:** 10.3762/bjoc.19.7

**Published:** 2023-01-24

**Authors:** Max Petitjean, José Ramón Isasi

**Affiliations:** 1 Department of Chemistry. University of Navarra. 31080 Pamplona, Spain

**Keywords:** chitosan, cyclodextrin polymers, green synthesis, locust bean gum, saponin, sorption, xanthan gum

## Abstract

Cyclodextrins, cyclic oligosaccharides with a hydrophobic cavity that form inclusion complexes with nonpolar molecules, can be used to functionalize other polysaccharides. Xanthan gum, locust bean gum or chitosan can be crosslinked using citric acid in the presence of β-cyclodextrin to produce insoluble matrices. In this work, polymeric foams based on those polysaccharides and saponin have been prepared using a green synthesis method to increase the porosity of the matrices. The saponin of soapbark (*Quillaja saponaria*) has been used to obtain foams using different procedures. The influence of the synthesis path on the porosity of the materials and their corresponding sorption capacities in the aqueous phase were evaluated.

## Introduction

Saponins are a family of natural molecules consisting of a hydrophobic aglycone backbone grafted with hydrophilic sugar molecules, allowing the plant to be protected from illnesses [1] and from herbivores endangerment [2]. The aglycone part is composed of steroid and triterpene molecules [3]. Not only present in plants [4–5], saponins have also been discovered in marine animals, such as sea cucumbers [6] or starfish [7]. Chemical structures of this family are varied [1], so they will show different properties [8]. Saponins, because of their amphiphilic nature, are known as natural surfactants [9]. They have been used as natural detergents, foaming agents, stabilizers, emulsifiers and wetting agents, for example [10]. The micelles produced will be different in size and shape as a function of the type of saponin, their aglycone forms but also the number of sugar molecules involved [11]. They can be found in beverage emulsions [12], and as food surfactants [13], because they are useful also to prevent the development of virus or bacteria in food or beverages [14–15]. As for medical purposes, it has been reported that saponins possess anticancer properties, by limiting proliferation and metastasis. This has been tested on different cancers such as leukemia [16], breast cancer [17] or prostate cancer to cite only a few of them [18]. They also present antimicrobial, antioxidant, anti-inflammatory, antidiabetic and cholesterol lowering properties, for example [10]. As reported by Liu et al. [19], saponins can show interesting interactions with hydrophobic organic compounds (HOC) and more precisely with polycyclic aromatic hydrocarbons (PAHs). For example, phenanthrene can be removed by saponins [20], by complexing with the PAH and having repulsive interactions with soil [21]. A large quantity of HOCs have been studied, such as naphthalene or fluoranthene [19]. The authors reported also the possibility of remediation of heavy metals by saponins [22]. Therefore, saponins are useful in soil washing technologies [23] or phytoremediation [24].

When chitosan and saponins are mixed, their foamability properties change, leading to a longer foam stability due to the higher viscosity achieved [25]. The use of chitosan to absorb saponins have been studied for different purposes. A high specific area activated carbon has been developed by Ma et al. [26], by the production of chitosan–saponin gels thanks to glutaraldehyde crosslinking. After adding potassium hydroxide, they freeze-dried the material and pyrolyzed the product. The resulting material possess a high absorption capacity of methylene blue, thanks to the presence of porosity and some chemical functions after pyrolysis. Chitosan–saponin–bentonite composite films can also be produced to absorb methyl orange and Cr(VI) [27]. Native [28] or derivatized [29] chitosans allow also a good sorption of *Quillaja* saponins useful to liberate them from wound dressings. This saponin possesses two large hydrophilic parts, surrounding the aglycone [30]. The association chitosan–saponin can show other functions for medicinal purposes, and anticancer nanoparticles made of chitosan loaded with saponins have been prepared by Nair and Jayakumar [31], and even coronavirus vaccine chitosan–saponin coatings have been developed to study its immunogenic potential [32].

A complexation between saponin and cyclodextrins (native or derivative) is possible [33], and the resulting release kinetics is appropriate for the creation of new saponin-based drugs [34]. Their potential uses can be either for oral delivery targeting intestine [33] or for an anti-skin cancer treatment [35], for example. This complexation step is also interesting for the synthesis of molecular imprinted polymers containing cyclodextrin [36].

In previous works, we have produced crosslinked polysaccharide networks using cyclodextrin to prepare green adsorbents [37]. The use of saponins added into the reactive mixture in order to produce a foam allows us to prepare porous materials, in order to enhance their sorption capabilities when a low amount of the sorbate is present in the solution. The specific area of the matrix is intended to be increased, permitting a greater accessibility to β-cyclodextrin sites.

## Results and Discussion

### Production of saponin foams

**Figure 1:**
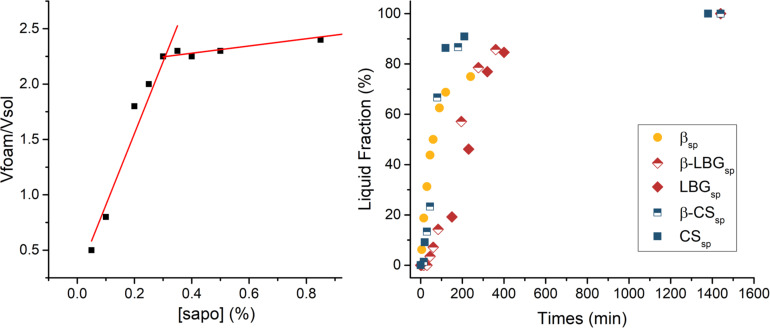
*Quillaja* saponin foamability (left) and foam stability over time for the β-cyclodextrin/polysaccharide (CS: chitosan, LBG: locust bean gum) mixtures (right).

As a first step, the determination of the foamability of saponin aqueous solutions allows us to find the surfactant concentration for which a maximum of foam volume will be produced. This value will correspond to the minimum amount of this ingredient required to produce the cyclodextrin/polysaccharide foams. As can be seen in Figure 1 (left), two linear fittings were applied in the foam volume vs concentration plot, where the junction of both lines gives us an approximate optimal foamability for a saponin concentration value of ca. 0.3%. These measurements correspond to solutions prepared using deionized water. Other factors such as the viscosity due to the polysaccharides or the ionic strength contributed by the catalyst can affect this value. Nevertheless, a concentration of 0.5% of saponin was selected as an initial value to prepare the polysaccharide foams.

In order to study their stability, the foams were introduced into a graduated cylinder after stirring each mixture solution (Figure 1, right). Samples with three different polysaccharides (chitosan (CS), locust bean gum (LBG) and xanthan gum (XG)) were tested either by themselves or mixed in a 50:50 ratio with β-cyclodextrin. A fast emergence of the liquid fraction occurred with the solution containing cyclodextrin with no polysaccharides. These results show that the emulsion production needs to be followed quickly by the freezing of the foam, otherwise a continuous liquid phase could be formed prior to the lyophilization process. The two chitosan solutions, with or without cyclodextrin, produce foams with similar stabilities. On the other hand, the presence of LBG impacts the stability of the foam in a remarkable way. Finally, the solutions of xanthan are not shown in Figure 1, because their foams are very stable due to the much higher viscosity of those solutions. In fact, the liquid fraction was not even falling down, and liquid agglomeration began to be observable at different heights in the graduated cylinder. Several interaction processes can influence the viscosity behaviour of these mixtures and have an impact on their stability. For instance, when considering mixtures of chitosan with saponin, the possibility of solubilization of chitosan molecules into the cavity of the glycoside micelles should be taken into account. Our main goal in this part of the study is to guarantee that the prepared foams remain stable at least until the crosslinking reaction takes place.

Once the six experimental synthetic paths were set (see Experimental section and Table 1), three types of matrices produced using saponin (*45spPow*, 45spLiq*, 45spFoam*) were compared to three with no saponin (*20Pow*, *45Pow*, 45Liq*). First of all, the synthesis procedure influences the yield achieved for each polysaccharide (Supporting Information File 1, Figure S1). In the absence of saponin, by increasing the reaction time from 20 to 45 min (samples *20Pow* and *45Pow*, respectively), the yield will increase due to a higher crosslinking efficiency of the solventless procedure [38]. A mixture dissolved in water and then freeze-dried (sample 45Liq*) shows a better yield, certainly because of a higher homogenization of the pre-crosslinked matrix. When prepared in powder (solid-state) form (*45spPow*), adding saponin will decrease the percent yield from 70% to 50%. Interestingly, the yield is not affected in that way when adding saponin into the liquid mixture (45spLiq*). However, crosslinking the foam (45spFoam*) will reduce considerably the yield for the chitosan matrix while the two other polysaccharides show no important modifications, which can be explained by the different stabilities of the foams produced.

**Table 1:** Methods of preparation of the thermally crosslinked cyclodextrin/polysaccharide matrices (*lyophilized).

Name	Crosslinking time (min)	Physical form	Saponin added

45spFoam*	45	foam	yes
45spLiq*	45	liquid	yes
45Liq*	45	liquid	no
*45spPow*	45	powder	yes
*45Pow*	45	powder	no
*20Pow*	20	powder	no

On the second set of yield results (Supporting Information File 1, Figure S1, bottom), the crosslinking of cyclodextrin with or without the polysaccharides using saponin show a slightly higher yield when prepared by lyophilization from the homogeneous liquid state (45spLiq*) than when lyophilized from the foam-like state (45spFoam*). These differences can be correlated to the foam stability.

### Morphology of the matrices studied by scanning electron microscopy (SEM)

**Figure 2:**
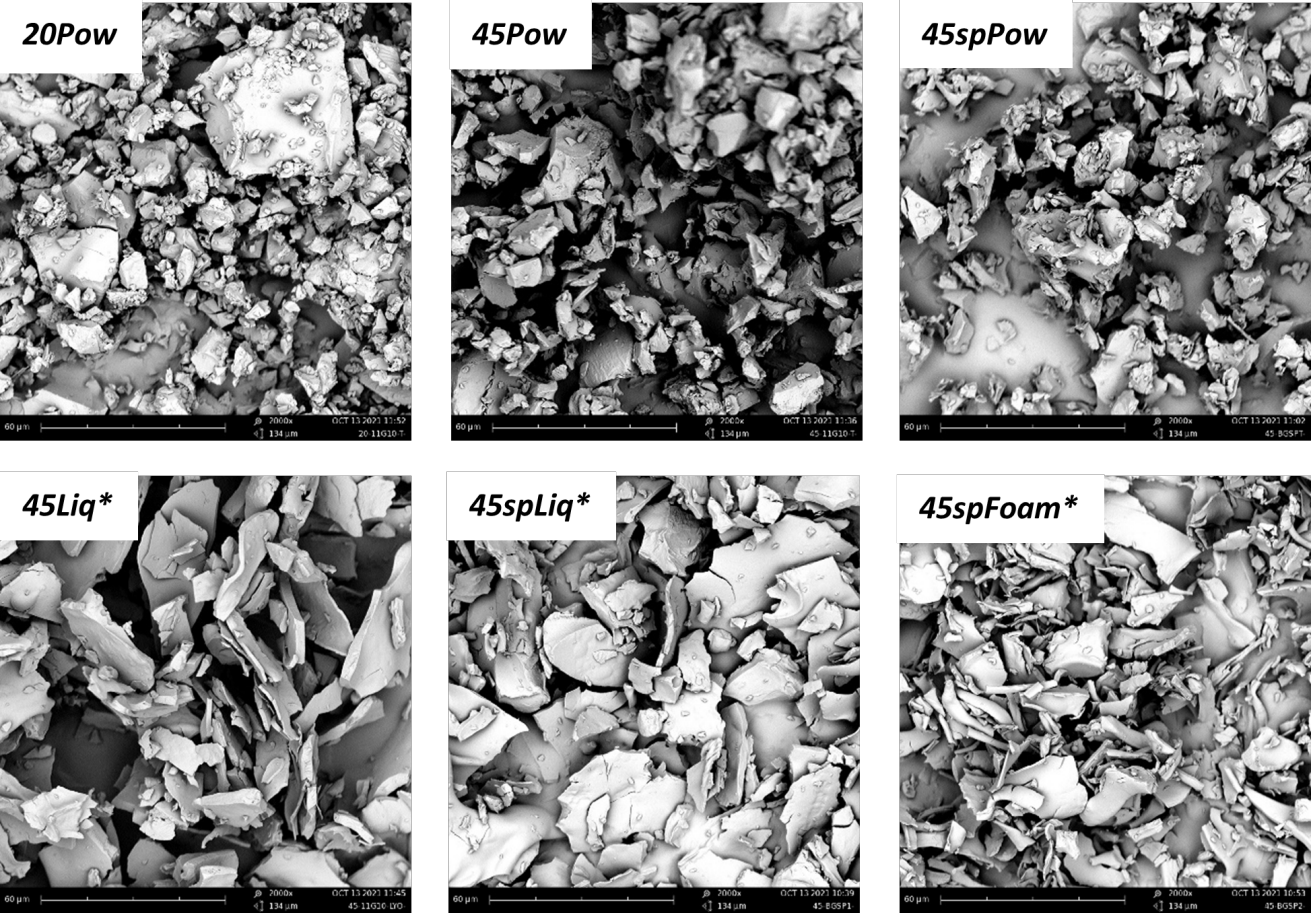
SEM images of crushed β-c-LBG as a function of the synthesis pathways (see below, Experimental section). Part “45Pow” of Figure 2 was reprinted from [39], Carbohydrate Polymers, vol. 288, by M. Petitjean; N. Lamberto; A. Zornoza; J. R. Isasi, “Green synthesis and chemometric characterization of hydrophobic xanthan matrices: Interactions with phenolic compounds“, article no. 119387, Copyright 2022 The Authors, with permission from Elsevier. Published by Elsevier Ltd, distributed under the terms of the Creative Commons Attribution-NonCommercial-NoDerivatives 4.0 International License, https://creativecommons.org/licenses/by-nc-nd/4.0/. This content is not subject to CC BY 4.0.

**Figure 3:**
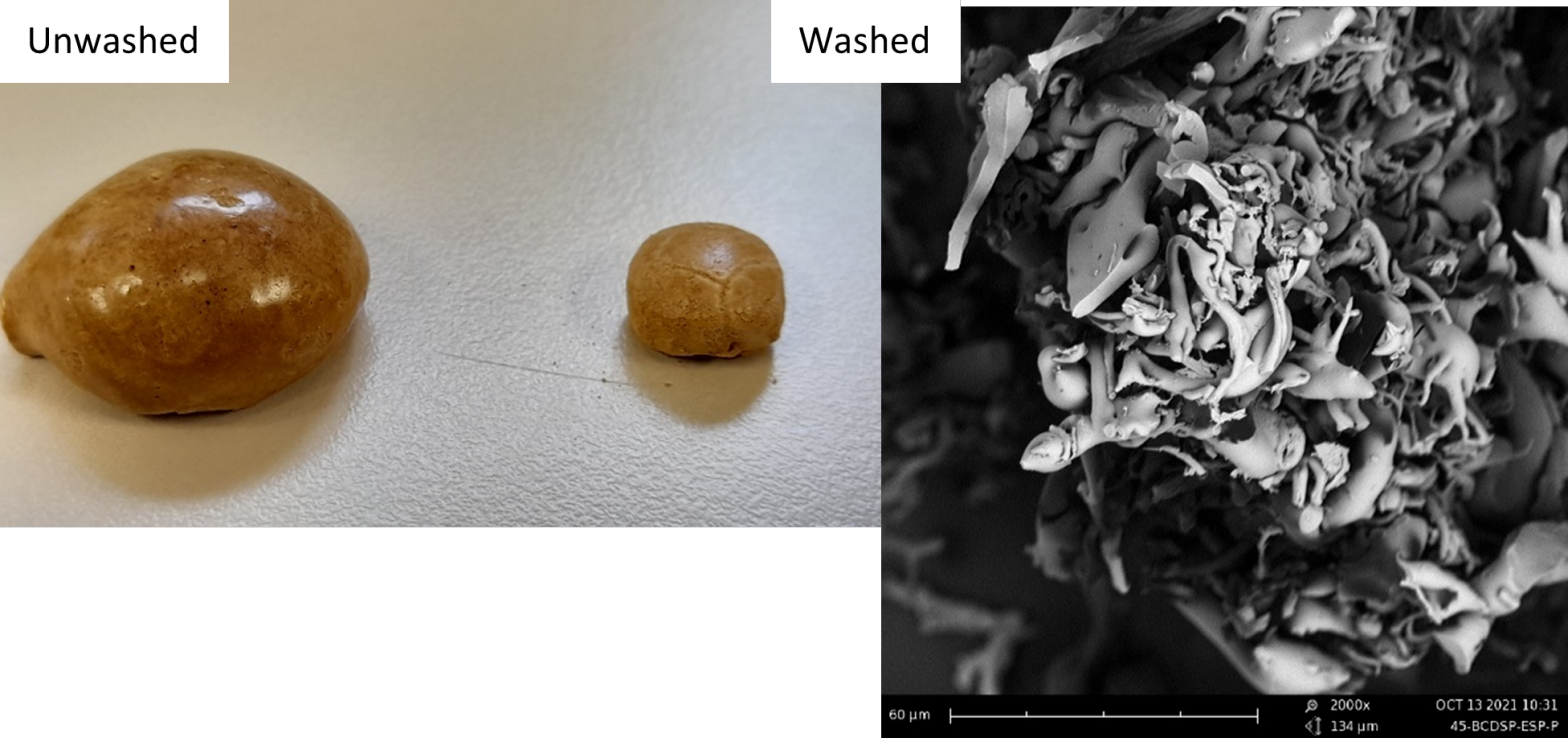
SEM of β-c_sp_ after crosslinking with or without washing the sample.

**Figure 4:**
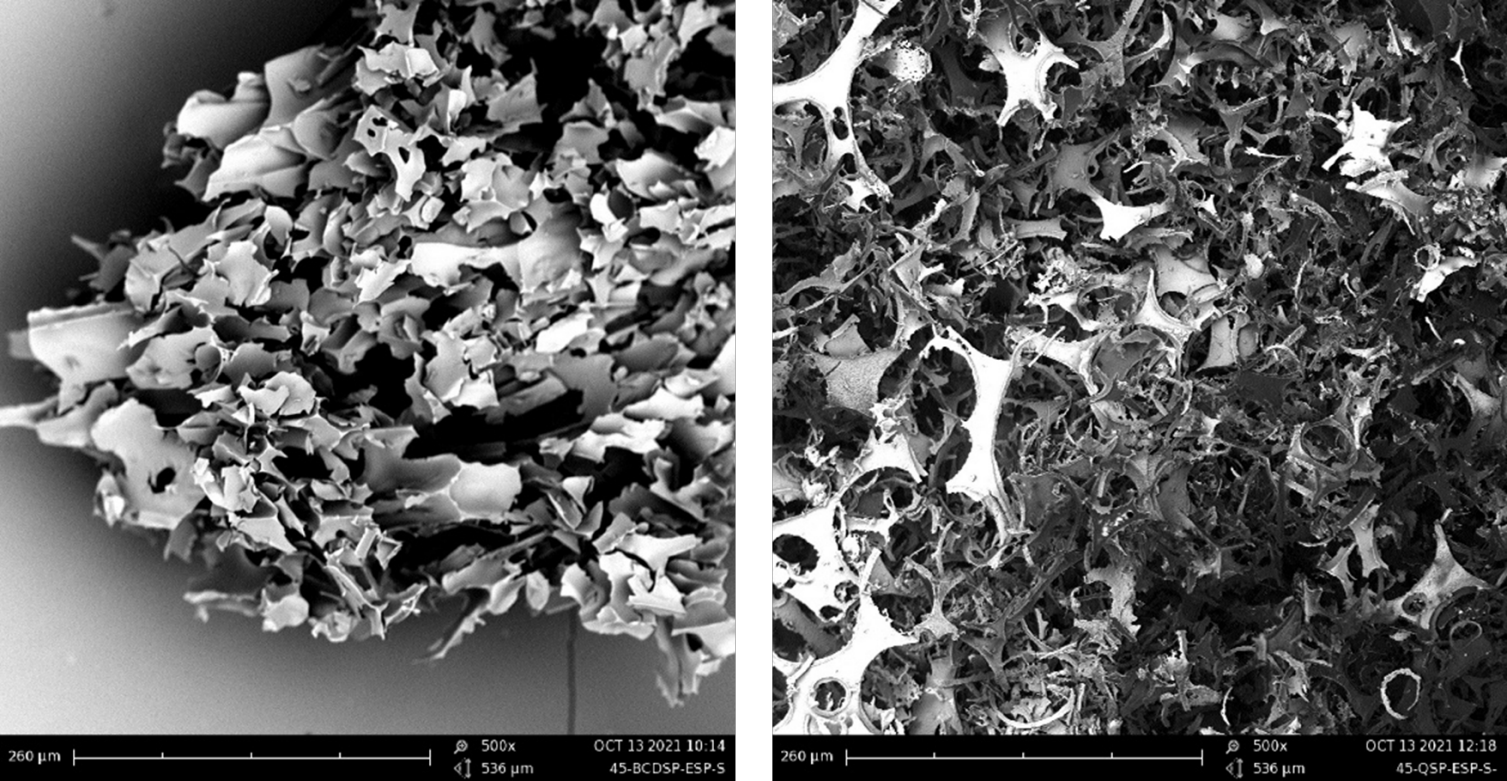
β-c_sp_ (left) and c-CS_sp_ (right) matrices unwashed showing the “foam-like” morphologies.

The different microstructures of the samples have been analysed for the three polysaccharide combinations. Those of β-cyclodextrin/LBG matrices will be shown here, since all the polysaccharides originated similar morphologies. In addition, the differences found for the three matrices prepared using the solventless procedures (*20Pow*, *45Pow*, *45spPow*) are not significant either. Figure 2 shows a powder-like material with the same average size (≈15 µm); some dispersity is detected in the *20 Pow* micrograph. In contrast, the use of the freeze-drying method produces some thinner and longer sheet-like particles. These sheets look also the same with or without the presence of saponin. The foaming process (spFoam*) creates thinner sheets than the liquid processes, and we can observe also some tubes for the latter, looking like sheets being rolled around themselves. These images correspond to the lyophilized, crushed, washed, and subsequently dried matrices. The washing process of the crosslinked matrices produces a swelling phenomenon because of the hydrophilicity of the polysaccharides and the citrate crosslinker, changing the morphology of the internal structures. As can be seen in Figure 3 for sample β-c_sp_ (cyclodextrin/saponin without polysaccharides), the washing process causes a swelling of the walls, transforming an ordered porous structure into a random structure, composed of a mixture of tubes and sheets. For these particular samples, the freeze-dried mixtures produce a spherical material in the heating step, covered by a fragile and brilliant layer but possessing a highly porous inner structure. This thin layer is more evident for the β-c_sp_ sample produced using the liquid path than for the one obtained by the foam path. The latter possesses also a higher specific area (Supporting Information File 1, Figure S2). On the other hand, the polysaccharide matrices (see Figure 4 for chitosan matrices) do no create the same type of pores; the successful emulsion process keeps its structure as shown by the spherical bubble pores. Chitosan produces fragile matrices once they are dried. However, looking at the structures produced by the xanthan gum and locust bean gum matrices, the presence of a sphere-like porous scaffold is also evident when the material is not washed.

### Chemical characterization of the β-cyclodextrin/saponin foams

The infrared spectra for the three crosslinked polysaccharides produced following different paths have been compared (Supporting Information File 1, Figure S3). In the fingerprint region, the main differences between the powder and liquid/foam matrices correspond to the ca. 1200 cm^−1^ region. The latter show a better resolution for the 1200 and 1150 cm^−1^ bands. In addition, a larger band at 1600 cm^−1^ is also observed for the saponin matrices; unfortunately, it is overlapped by other bands present in the polysaccharide spectra, so the amount of saponin incorporated into the matrices is difficult to quantify by this method. The infrared study of this region for similar samples (in the absence of saponin) has been reported in our previous works [37–39]. In addition to those, a small band can be detected also around 1500 cm^−1^ only for saponin matrices. This one might be useful for quantification purposes, provided some validation can be obtained using other appropriate methodologies.

A shift of the 1000 cm^−1^ C–O band towards higher wavenumbers (Supporting Information File 1, Figure S4) occurs for each matrix when going from 100:0 to 0:100 ratio of cyclodextrin/polysaccharide. The 1200 cm^−1^ band is also more intense when changing that ratio. This difference is also correlated to the C=O band found at 1700 cm^−1^. In the case of the chitosan matrices, for a high concentration of chitosan, the crosslinking reactions include esterification links, amide formation and the Maillard reaction [39]. This behaviour can be analysed by the intensities of the 1600–1500 cm^−1^ regions.

**Figure 5:**
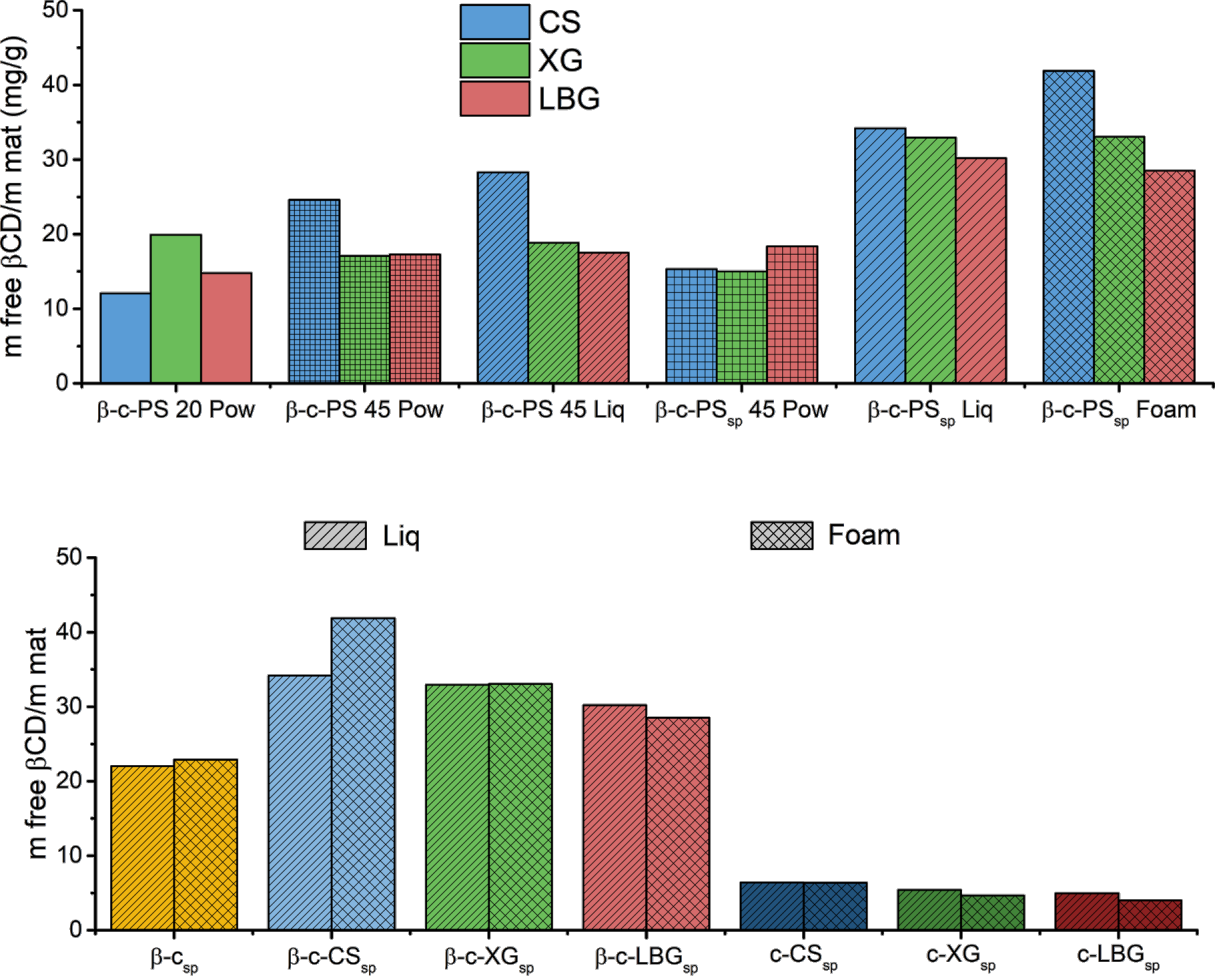
Values (in mg/g) of equivalent ‘free β-cyclodextrin’ in the polysaccharide (PS) matrices, as a function of the synthesis path (top), and comparisons between the liquid and foam saponin matrices with (bottom left) or without cyclodextrin (bottom right).

An interesting method of comparison from the molecular point of view is the use of phenolphthalein as a probe to analyse the amount of ‘free cyclodextrin’ moieties present in the matrices, i.e., those available for inclusional interactions [38]. The first and most important difference is between both liquid and foam saponin samples and the rest (Figure 5). The amount of ‘free cyclodextrin’ found for the solventless (powder) synthesis, with or without saponin, is always low, around 15 mg/g of matrix. Similar results are found for the liquid path in the absence of saponin. The favourable influence of saponin to produce a more efficient matrix is confirmed. The freeze drying of solutions permits to produce microporous materials, as seen in the SEM images of Figure 2, where the sheet-like structures observed being those macrostructures modified by water swelling and crushing after drying. Saponins, at a molecular level, probably confer an additional microporosity to the matrix, permitting the cyclodextrin moieties to be more available for complexation with phenolphthalein or other molecules.

In addition, the foam and liquid saponin/cyclodextrin/polysaccharide samples are compared to the corresponding cyclodextrin/saponin (no polysaccharide) and saponin/polysaccharide (no cyclodextrin) matrices (Figure 5, bottom). All the cyclodextrin/polysaccharide samples show a higher amount of ‘free cyclodextrin’ per gram than that of pure cyclodextrin. This may be due to a possible saponin/β-CD complexation, yielding the cyclodextrin unavailable, or because the polysaccharides enhance the grafting of cyclodextrin into their chains. It needs to be added that pure polysaccharide matrices (last columns in Figure 5, bottom) show also an affinity with phenolphthalein. Because these samples do not possess any cyclodextrin modifications, this non-zero values should be explained as an ‘equivalent free cyclodextrin’. In our previous work [38], we checked that those crosslinked polysaccharide matrices without cyclodextrin did not show any affinities towards phenolphthalein, so, in this case, we can attribute this effect to certain favourable interactions between phenolphthalein and the saponin moieties.

### Sorption capabilities of the matrices

**Figure 6:**
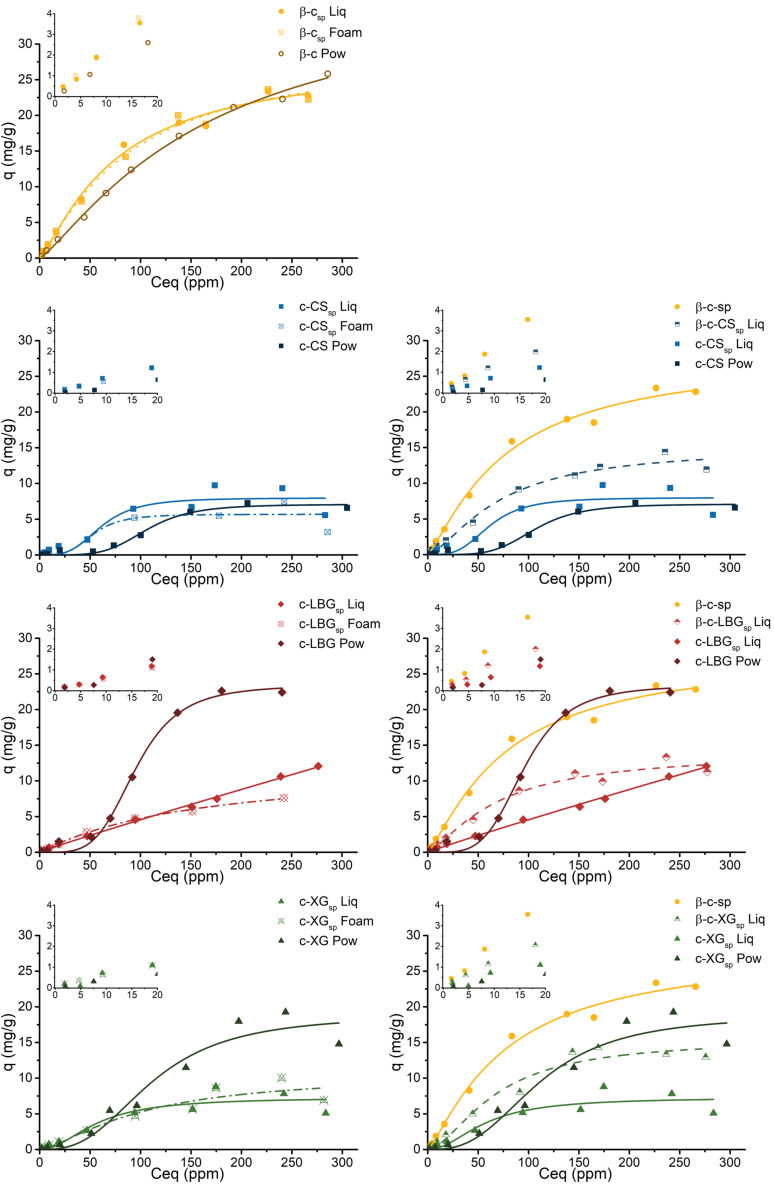
1-Naphthol isotherms of crosslinked β-cyclodextrin/polysaccharides (blue curves for chitosan, red for locust bean gum, green for xanthan gum) with saponin (yellow curves correspond to cyclodextrin matrices, without polysaccharide).

This ‘free cyclodextrin’ impact on the sorption capabilities of the matrices has been studied with the corresponding 1-naphthol (1-N) isotherms (Figure 6). When comparing the isotherms of *20Pow*, *45spLiq* and *45spFoam* of single component matrices, a better sorption from the saponin matrices for low 1-naphthol concentrations is detected. Thus, β-c_sp_ (with no polysaccharide) absorbed more 1-N when saponin is present between 2 ppm and 200 ppm, which is the largest range of higher efficiency for all matrices. For the others, there seems to be no effect in the low 1-N concentration range. Nevertheless, we need to look at the graph insets, which show the sorption isotherms at low solute concentrations. The c-LBG *20Pow* matrix sorption behaviour was fitted following the Hill function, but this model does not represent well the absorption process in the low concentration region. For c-XG and c-CS, a wider 1-N concentration range where saponins impact on sorption is observable: between 2 and 50 ppm for c-XG, and between 2 and 150 ppm for c-CS. As a possible explanation for this anomalous behaviour, it is known that 1-N can self-associate from a certain concentration level. Before this, the 1-N molecules are easily complexed within cyclodextrin matrices, or, in this case, associated to the new hydrophobic regions provided by saponin. When this concentration is higher, the association of 1-N appears to create aggregates, saturating the cyclodextrin sites but allowing the polysaccharide networks affect the sorption. When saponin is introduced, this last impact can be assumed by the saponin moieties. The difference between *45spLiq* and *45spFoam* is quite small; it is not observable for β-c_sp_, and the biggest difference is seen for c-LBG_sp_ produced by a “liquid path” synthesis. This variation reflects the sorption mechanism, as the curve follows the Freundlich model, while the Hill model represent the others better. As seen for the isotherms in previous works, the higher the cyclodextrin/polysaccharide ratio, the better the sorption. The effects of LBG or XG on the sorption is absent when saponin is in the matrix, letting these polysaccharides being a simple hydrogel scaffold for the sorption of 1-naphthol.

**Figure 7:**
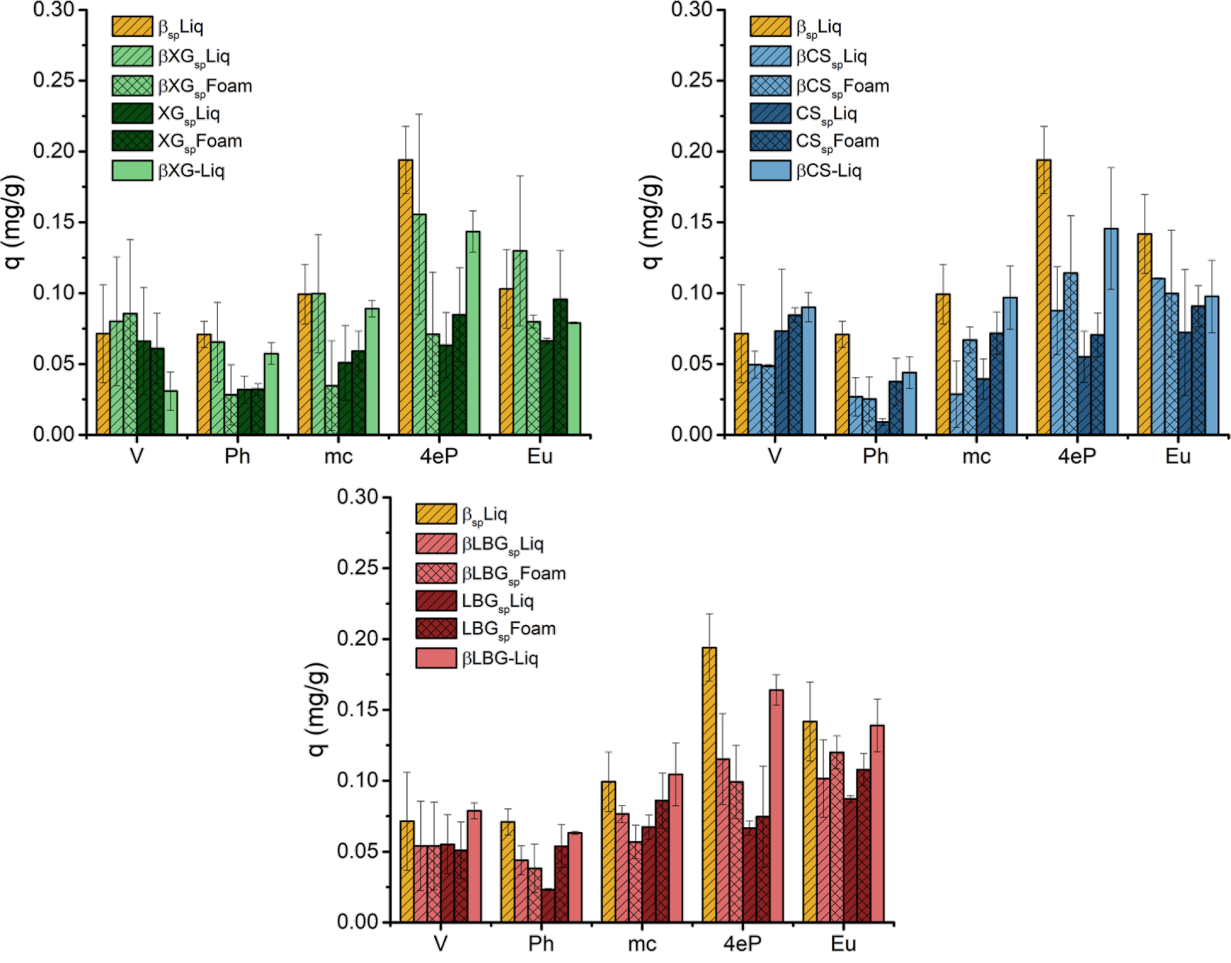
Sorption of phenols (V, vanillin; Ph, phenol; m-c, *m*-cresol; 4eP, 4-ethylphenol; Eu, eugenol) in β-cyclodextrin/polysaccharides (xanthan, XG; chitosan, CS; and locust bean gum, LBG) with saponin (sp) produced by different pathways (liquid and foam).

An aqueous sorbate mixture of five phenolic compounds has been also tested to assess the differences in the sorption behaviours of the different matrices. The absorbed amount changes as a function of the cyclodextrin/polysaccharide ratio, and the same trends are observed for all the polysaccharide types (Figure 7). Those matrices with cyclodextrin (yellow bars in Figure 7) absorbed better all the phenolic compounds tested. In addition, the incorporation of saponin does not produce a higher sorption capacity except for the XG matrices. In these matrices, the sorption of 4-ethylphenol and eugenol are the most favourable, followed by that of vanillin. Finally, the synthesis pathway (either liquid or foam) is not a determinant factor for the sorption capacity either. This fact can be explained by the similar microstructures produced in each case, though their macroporosities are remarkably different.

## Experimental

### Materials

β-cyclodextrin (Wacker, 12.5% H_2_O), xanthan gum (Sigma-Aldrich), locust bean gum (Sigma-Aldrich), chitosan (deacetylation degree of 90%), citric acid (Panreac AppliChem) and dibasic sodium phosphate (Na_2_HPO_4_ ≥ 98%), soapbark (*Quillaja saponaria*; Sp. ‘quillay’, from Mapuche ‘küllay’) saponin (Sigma-Aldrich, sapogenin content ≥ 10%, India), phenol (99.5%; Panreac, Spain), *m*-cresol (99%; Sigma, Germany), 4-ethylphenol (99%, Sigma, China), vanillin (99%; Panreac, Spain) and eugenol (99%; Sigma, Germany) were used as received. Phenolphthalein and 1-naphthol (≥99%) were obtained from Merck (Germany).

### Methods

**Synthesis procedures:** Firstly, citric acid (1.3 g) is dissolved into 100 mL of deionized water (acidic pH is required for chitosan) with 1.5 g of polysaccharide (either xanthan, or locust bean gum, or chitosan) and/or β-cyclodextrin, plus 0.5 g of *Quillaja saponaria* saponin. After that, sodium phosphate dibasic (Na_2_HPO_4_, 0.28 g) is added to catalyse the reaction (see Supporting Information File 1, Table S1).

Two main types of materials are prepared from these stock solutions. The first one (45spLiq*) is directly lyophilized until a perfectly dried sample is obtained (two freeze drying steps of 24 h can be necessary with an additional freezing at −40 °C between the two). The resulting material is then thermally crosslinked at 170 °C during 45 min. Then, it is washed twice in 100 mL of deionized water, filtered and freeze dried again. The second process (45spFoam*) consists of introducing 20 mL of the stock solution into a large crystallizer, where the liquid height reaches around 1 cm. The liquid is stirred during 5 min at high speed to make an air/water emulsion. The resulting foam is quickly frozen at −40 °C, lyophilized, thermally crosslinked, washed and dried using the same conditions as for the previous path.

**Figure 8:**
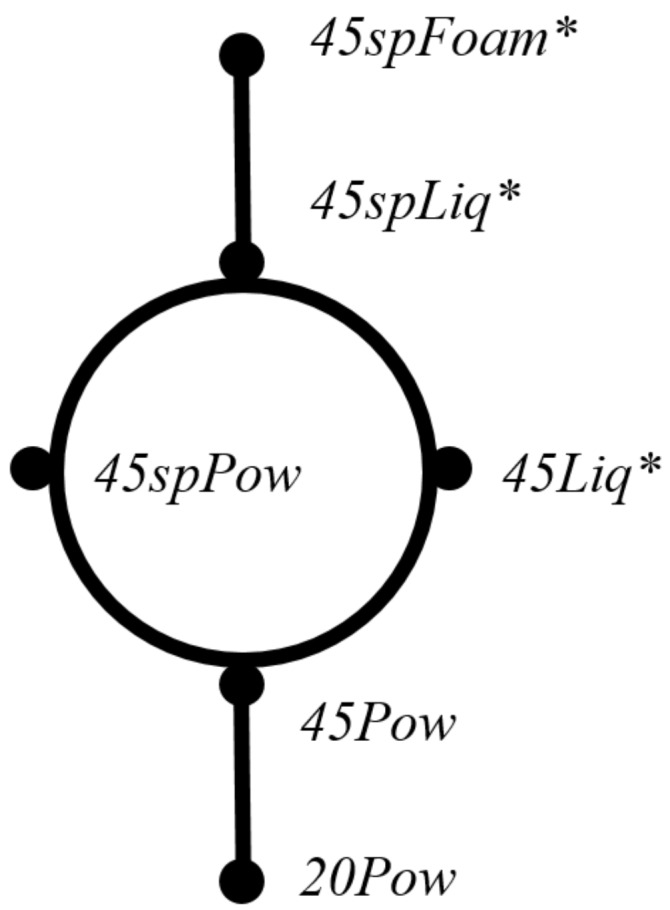
Six synthesis routes (*lyophilized matrices) used to prepare samples β-c-XG_sp_; β-c-LBG_sp_; β-c-CS_sp_ using β-cyclodextrin (β), citric acid (c), xanthan gum (XG), locust bean gum (LBG) and chitosan (CS) with saponin (sp) in different physical forms (see also Table 1).

In order to understand the effect of saponins and the influence of the synthesis procedures, the crosslinked matrices have been prepared by four other routes, with or without saponin, starting either from solutions or in the solid state, as shown in Figure 8. Thus, the solid mixtures were homogenized using a mortar as in previous works [38–39], and crosslinked at 170 °C during 20 or 45 min (samples *20Pow* and *45Pow*). A solid-state mixture with the addition of 0.5 g of saponin and a thermal crosslinking at 170 °C during 45 min was prepared also, for comparison purposes (sample *45spPow*).

Finally, the three reagents (cyclodextrin, polysaccharide and citric acid) plus the catalyst, without saponin, were dissolved into 100 mL of deionized water, freeze dried, crosslinked, washed and dried as for the first path explained in this section (sample 45Liq*). The matrices formed were then crushed during 30 seconds in a Retsch MM300 ball mill.

**Saponin solutions:** The foamability of *Quillaja* saponin was measured by dissolving different concentrations of saponin into deionized water (from 0.05 to 1%). Then, 20 mL of the solutions were vigorously agitated with a mechanical stirrer. The maximal foam volume is then measured and plotted against the saponin concentration (see Figure 1, above). To measure the foam stability, the foam was recovered and introduced into a graduated cylinder. The liquid volume was measured at different times and the percent emergence of the liquid fraction was plotted vs time (see also Figure 1).

**Characterization of matrices:** Scanning electron microscopy (SEM) of gold-sputter-coated samples was carried out using a Phenom Pro 739 microscope. Infrared analysis was carried out for the crushed samples using a Shimadzu IRAffinity-1S instrument coupled with a Golden Gate^™^ attenuated total reflectance (ATR) accessory device (Specac).

**Sorption experiments:** The absorption of phenolphthalein and 1-naphthol was analysed using an Agilent Technologies Cary 8454 UV–vis device, equipped with an Agilent ChemStation software. In the case of the phenolic mixtures, an Agilent 110 series HPLC system with a Phenomenex Luna C18 column and a gradient mobile phase were used (H_2_O 65% to 50%, acetonitrile 25% to 40%, plus 10% methanol).

## Supporting Information

File 1 Percent yields reached following different synthetic paths, additional SEM micrographs, infrared spectra of the samples in the fingerprint region, table with compositions of the reacting mixtures.
