# Supplementary material for: Preparation of β-cyclodextrin/polysaccharide foams using saponin
Source: Beilstein J Org Chem. 2023 Jan 24;19:78–88. doi: 10.3762/bjoc.19.7 (PMC9887783; doi:10.3762/bjoc.19.7)
Supplement: File 1 — Percent yields reached following different synthetic paths, additional SEM micrographs, infrared spectra of the samples in the fingerprint region, table with compositions of the reacting mixtures. [file Beilstein_J_Org_Chem-19-78-s001.pdf]

BEILSTEIN JOURNAL OF ORGANIC CHEMISTRY

Supporting Information

for

Preparation of β-cyclodextrin/polysaccharide foams using saponin

Max Petitjean and José Ramón Isasi

Beilstein J. Org. Chem. **2023,** *19,* 78–88. doi:10.3762/bjoc.19.7

Percent yields reached following different synthetic paths, additional SEM micrographs, infrared spectra of the samples in the fingerprint region, table with compositions of the reacting mixtures

License and Terms: This is a supporting information file under the terms of the Creative Commons Attribution License (https://creativecommons.org/licenses/by/4.0). Please note that the reuse, redistribution and reproduction in particular requires that the author(s) and source are credited and that individual graphics may be subject to special legal provisions.
The license is subject to the *Beilstein Journal of Organic Chemistry* terms and conditions: (https://www.beilstein-journals.org/bjoc/terms)

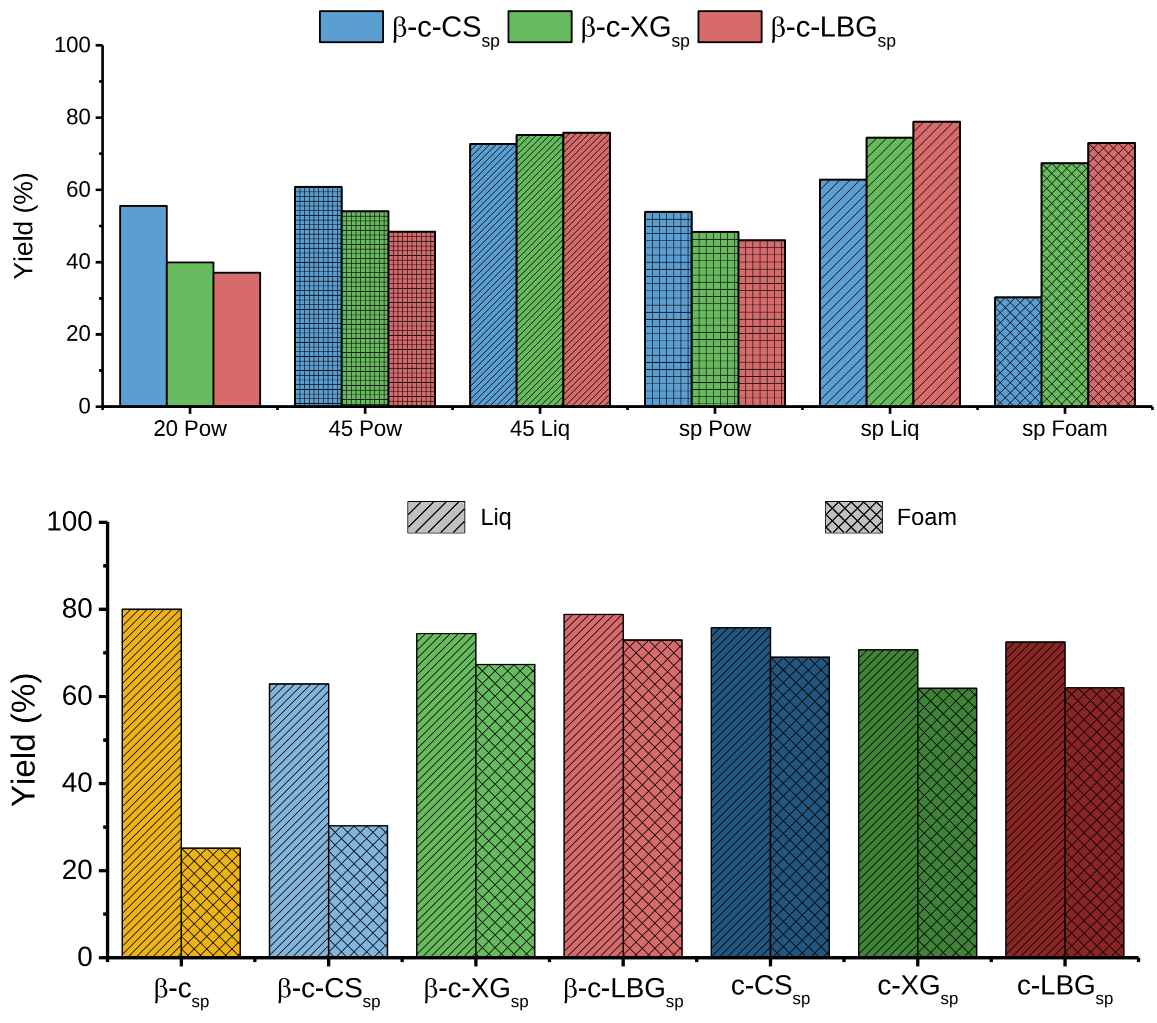

Figure S1. Percent yields reached following different synthetic paths (top) and comparison of the last two methods prepared using different CD:PS ratios (bottom).

Unwashed

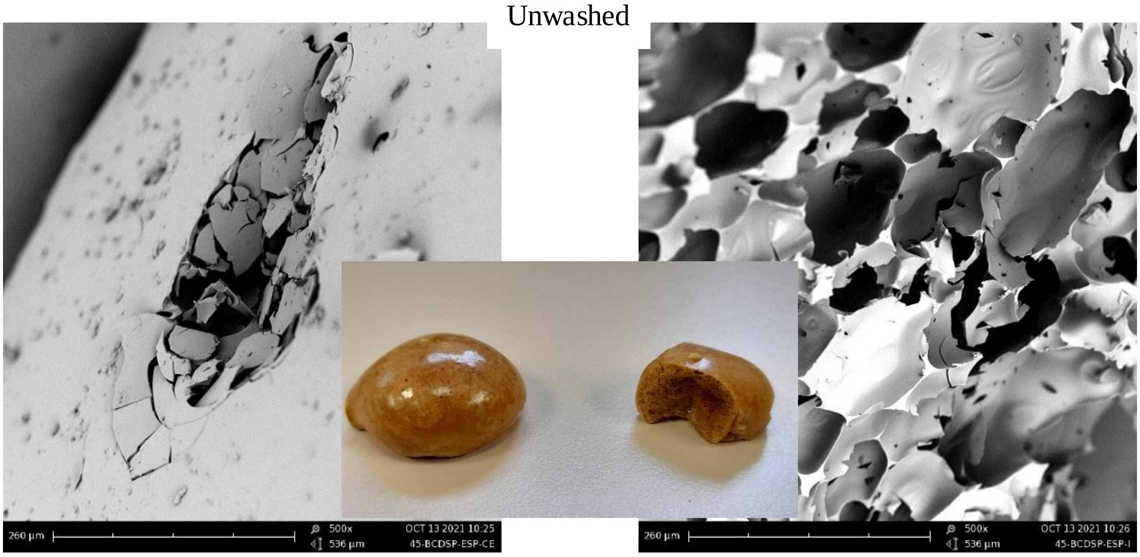

Figure S2. SEM of β-c_{sp} matrix unwashed showing outer (left) and inner(right) micrographs.

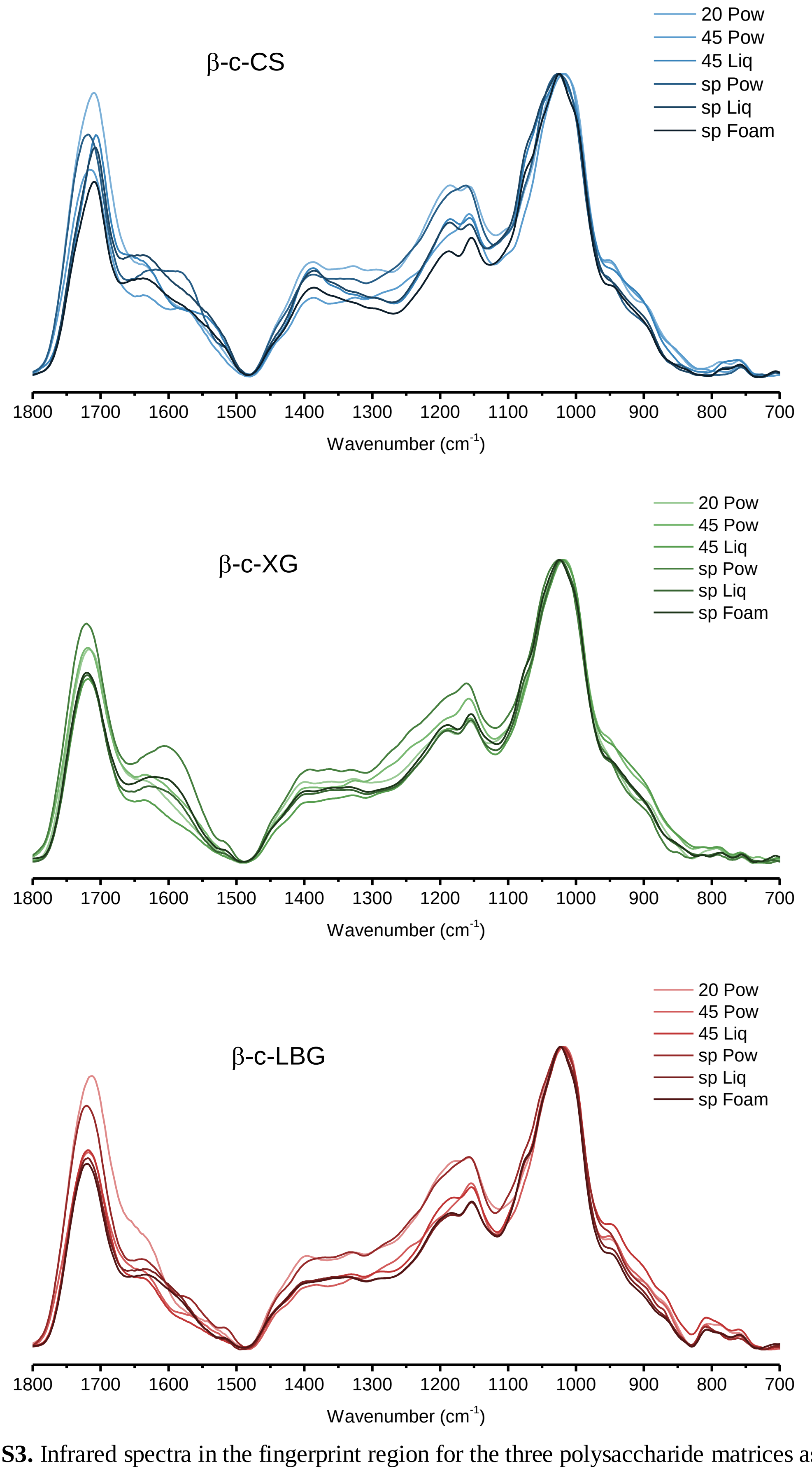

Figure S3. Infrared spectra in the fingerprint region for the three polysaccharide matrices as a function of the preparation method.

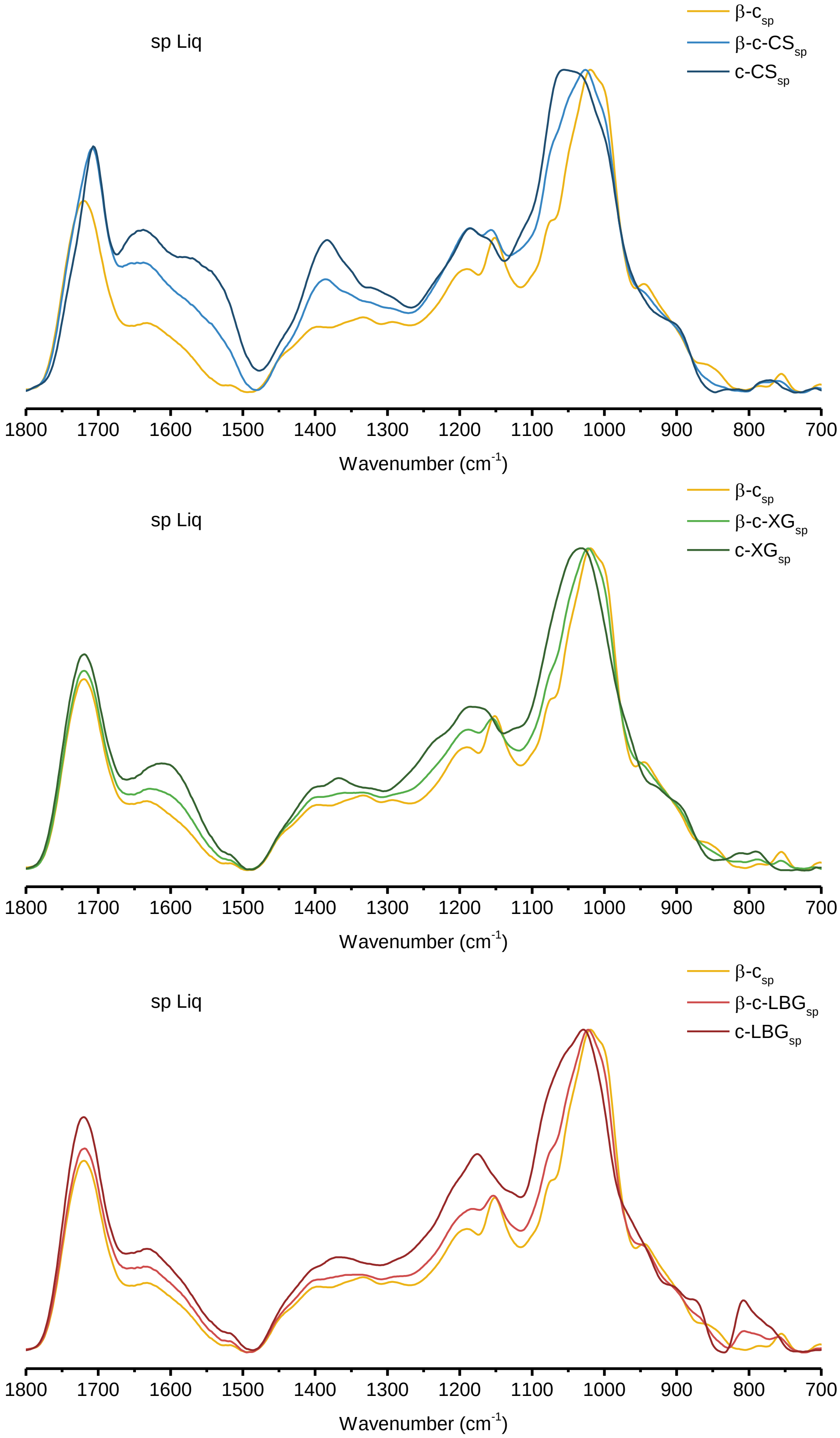

Figure S4. Infrared spectra in the fingerprint region for matrices prepared using saponin by the "liquid path" for pure crosslinked cyclodextrin compared to the three polysaccharides with or without cyclodextrin.

Table S1. Amounts of components (in g) used to prepare saponin crosslinked matrices.

Names	CTR	XG	LBG	CS	βCD	Sapo	Na_2HPO_4
**c-XG$_{sp}$**	1.3	1.5	/	/	/	0.5	0.28
**c-LBG$_{sp}$**	1.3	/	1.5	/	/	0.5	0.28
**c-CS$_{sp}$**	1.3	/	/	1.5	/	0.5	0.28
**β-c-XG$_{sp}$**	1.3	0.75	/	/	0.75	0.5	0.28
**β-c-LBG$_{sp}$**	1.3	/	0.75	/	0.75	0.5	0.28
**β-c-CS$_{sp}$**	1.3	/	/	0.75	0.75	0.5	0.28
**β-c$_{sp}$**	1.3	/	/	/	1.5	0.5	0.28
